# Compound-Resolved VOC Dynamics in a Full-Scale Medium-Density Fibreboard Dryer: Process–State Screening Across Wood Furnish, Amino Resin Dosing, and Thermal Operating Variables

**DOI:** 10.3390/polym18101230

**Published:** 2026-05-18

**Authors:** Vladimir Nedić, Andreas Paul, Marius Catalin Barbu, Lubos Kristak

**Affiliations:** 1 Faculty of Wood Sciences and Technology, Technical University in Zvolen, T. G. Masaryka 24, 960 01 Zvolen, Slovakia; vladimir.nedic@swisskrono.com; 2 SWISS KRONO TEX GmbH & Co. KG, Wittstocker Chaussee 1, 16909 Heiligengrabe, Germany; andreas.paul@swisskrono.com; 3 Design and Green Engineering Department, Salzburg University of Applied Sciences, Markt 136a, 5431 Kuchl, Austria; marius.barbu@fh-salzburg.ac.at; 4 Faculty of Furniture Design and Wood Engineering, Transylvania University of Brasov, Bdul. Eroilor nr. 29, 500036 Brasov, Romania

**Keywords:** medium-density fibreboard, volatile organic compounds, industrial dryer, formaldehyde, terpenes, amino resin adhesive, process–state screening

## Abstract

Industrial control of volatile organic compound (VOC) emissions from medium-density fibreboard (MDF) production remains constrained by a shortage of compound-resolved evidence from full-scale plants, where wood furnish, amino resin chemistry, heat transfer, gas flow, and wet gas cleaning act simultaneously. Here, we analysed more than 20,000 synchronized operating records from a full-scale single-stage flash-tube MDF dryer at an industrial SWISS KRONO production line and linked total VOC (TVOC) measurements from flame ionization detection with Fourier-transform infrared speciation on the cleaned stack. Five compounds—α-pinene, 3-carene, limonene, methanol, and formaldehyde—accounted for more than 80% of the resolved VOC signal. Process–state contrasts showed that higher digester residence time, discharge screw speed, adhesive amount, urea amount, dryer inlet temperature, and scrubber–water temperature increased one or more representative compounds, whereas higher hardwood share, additional flue-gas supply, and higher scrubber–water pH decreased them. Limonene, methanol, and formaldehyde were substantially more process-sensitive than α-pinene. An exploratory decorrelation step further showed that a drying/throughput domain explained about half of the variability of the screened process space. The study therefore identifies the small set of compounds and operating domains that most strongly govern the cleaned dryer-stack signature and provides a mechanistically grounded prioritization framework for follow-up causal experiments, source apportionment, and emission-mitigation design in industrial MDF manufacture. Unlike product or chamber emission studies, this work links the compound-resolved FTIR/FID chemistry of the final cleaned industrial stack with synchronized production variables; it therefore addresses a scale-integration gap by transforming routine compliance-type exhaust monitoring into a process-diagnostic framework for ranking emission sources, abatement-sensitive variables, and mitigation experiments.

## 1. Introduction

Medium-density fibreboard (MDF) is among the most chemically coupled engineered wood products because dry-process manufacture forces lignocellulosic polymers, wood extractives, water, inorganic additives, and thermosetting amino resins to interact under rapidly changing hydrothermal and aerodynamic conditions [1,2,3,4]. In a commercial line, chips are steamed and defibrated, fibres are blended in the blowline with urea–formaldehyde (UF) or melamine–urea–formaldehyde (MUF) adhesive and auxiliary chemicals; the resulting fibre–resin aerosol is exposed to short-residence flash-drying before dust separation and end-of-pipe treatment. The measured dryer-stack signal is therefore not a simple property of the finished board or the raw wood; it is the chemically integrated outcome of biomass thermochemistry, polymer curing and hydrolysis, gas-particle transfer, and compound-selective abatement. From an emission science perspective, the MDF dryer should be treated as a reactive multiphase system rather than as a single emission source.

The main volatile classes observed in MDF-related systems arise from distinct but interacting origins. Monoterpenes such as α-pinene, β-pinene, 3-carene, and limonene are governed largely by wood species, extractive inventory, storage history, and the extent to which these native compounds survive cooking and drying. Methanol is associated mainly with cleavage and deacetylation reactions in hemicelluloses; aldehydes and organic acids reflect thermo-hydrolytic and thermo-oxidative conversion of wood polymers and extractives; and formaldehyde is influenced not only by native wood chemistry but also by residual-free formaldehyde, curing chemistry, hydrolysis, and thermal stress in UF and MUF adhesive systems [5,6,7,8,9,10,11,12,13,14,15,16]. Because these pathways overlap in time and space, any operating change that modifies furnish composition, steaming severity, resin addition, scavenger dosage, fibre temperature, residence time, or gas-cleaning conditions can alter both the magnitude and the chemical fingerprint of emitted VOCs.

This problem has both technological and regulatory relevance. On the product side, low-emission panel development has advanced substantially through reduced-UF formulations, scavenger strategies, lignin-containing binders, and hybrid bio-based adhesive concepts. Antov et al., for example, demonstrated that HDF manufactured from industrial hardwood fibres with only 3% UF resin and ammonium lignosulfonate can reach very low formaldehyde contents, in the range of 0.7–1.0 mg/100 g, while retaining acceptable board performance [17]. On the process side, however, emission control is increasingly driven by plant-level requirements. The German TA Luft framework and the European BAT reference document identify dryers, presses, and associated exhaust streams as critical sources of VOCs and formaldehyde in wood-based panel manufacture, making source apportionment and process–state control central industrial questions [18,19].

The available literature is informative but methodologically fragmented. Chamber and cell studies on finished boards have been indispensable for indoor-air assessment and for the derivation of formaldehyde and VOC emission parameters, while recent analytical reviews have clarified the benefits and limits of chambers, field and laboratory emission cells, perforator methods, TD-GC/MS, PTR-MS, and optical or online approaches for engineered wood products [6,7,11]. A second group of studies has focused on manufacturing-stage transitions. He et al. showed that the VOC composition of wood-based panels changes substantially from wood chip to resin-coated fibre to finished board, and that formaldehyde is dominated by the resin side of the system, whereas much of the broader VOC spectrum originates from the wood itself [13]. MDF-specific studies further showed that furnish composition, pulping route, and pressing conditions change the release of terpenes, aldehydes, volatile acids, and formaldehyde [9,10,14]. Carbonyl-focused analysis of dry-process fibreboards has recently expanded this picture by resolving formaldehyde, acetaldehyde, acetone, and higher aldehydes in commercial MDF samples [20], while PTR-MS studies have emphasized how wood species and finishing systems reshape VOC intensity and profile in commercial panels [21].

A third body of literature, stemming more from drying science and air pollution control than from product testing, demonstrates that emission behaviour is strongly conditioned by moisture content, temperature, hydrothermal severity, and treatment technology. Wood-drying studies have shown sharp increases in VOC or HAP release as the moisture content approaches critical thresholds and as thermal severity increases [22,23,24]. Complementary MDF-focused studies demonstrated that formaldehyde emission behaviour remains highly sensitive to temperature and humidity both in a full-scale experimental room and in controlled chamber tests, confirming that environmental and transport conditions substantially reshape the apparent emission response [25,26,27,28,29,30]. These findings are highly relevant to MDF dryer exhausts, because industrial measurements integrate source generation, convective transfer, and end-of-pipe conditioning simultaneously. Yet, a critical gap remains. Most published work either characterizes finished boards, isolates a single manufacturing stage, or examines untreated emissions before final abatement. Far fewer studies link compound-resolved stack chemistry to synchronized plant variables under full-scale commercial operation after scrubber treatment. That gap matters scientifically because the cleaned stack is the place where wood chemistry, amino resin chemistry, dryer operation, and abatement selectivity finally converge. The present study addresses this gap by analysing the cleaned exhaust of a full-scale MDF dryer operated under industrial conditions after wet scrubbing and biological wastewater treatment. The objectives were as follows: to identify the dominant FTIR-resolved compounds in the cleaned stack; to quantify the directional response of representative markers to screened process–state contrasts spanning furnish composition, hydrothermal severity, throughput-linked dosing, thermal load, and scrubber conditions; and to reduce the observed process interactions to a smaller set of mechanistically interpretable operational domains. The novelty of the work lies not in another chamber assessment of boards, but in the compound-resolved interpretation of a running industrial emission system in which wood polymers, extractives, amino resin binders, process control, and end-of-pipe treatments interact continuously.

The methodological novelty is therefore a scale-integrated process–emission analysis rather than another product–emission test. Earlier studies have provided essential knowledge on chamber emissions from finished boards, carbonyl or terpene profiles, adhesive-related formaldehyde release, and analytical approaches for engineered wood products; however, those designs generally decouple the emission source from the plant-control system and from end-of-pipe treatment. The present study couples four information layers that are rarely available in one dataset: (i) FID-based total-carbon tracking, (ii) FTIR-based molecular speciation, (iii) more than 20,000 synchronized records from a running MDF line, and (iv) interpretation of the post-scrubber cleaned stack, where source formation, gas-phase transport, wet absorption, and biological post-treatment have already interacted. This allows the paper to move from the question “which VOCs can MDF emit?” to the more industrially decisive question “which chemical markers and operating domains control the final regulated stack signature under full-scale production?”

The contribution is deliberately framed as a causal hypothesis and prioritization map. It does not overclaim that an observational campaign can replace an orthogonal experiment. Instead, it identifies the reduced set of emission markers and coupled operating domains that should be controlled, monitored, or deliberately varied in the next industrial factorial campaign. This is the central value of the work: it provides the missing bridge between laboratory emission chemistry and plant-scale emission-control engineering.

## 2. Materials and Methods

### 2.1. Industrial System and Study Design

The study was carried out during the first quarter of 2022 on an industrial MDF line operated by SWISS KRONO TEX GmbH & Co. KG in Heiligengrabe, Germany. The industrial emission-measurement campaign was documented by DIEFFENBACHER GmbH Maschinen- und Anlagenbau (Eppingen, Germany). The analysed system comprised a single-stage flash-tube fibre dryer supplied by secondary steam-heated registers, supplemental hot gas from the energy plant, and a gas-fired surface burner. The exhaust stream passed through a wet scrubber with biological wastewater post-treatment before release via the stack. Measurements were performed on the clean-gas side of the stack, i.e., after the integrated gas-cleaning step, so the reported values represent the final emission signature rather than raw dryer gas.

More than 20,000 synchronized plant records were available for evaluation. Because the production line was operated under commercial manufacturing conditions, the study design was observational: operating states were screened from historical process data and contrasted as lower-setting state A versus higher-setting state B. The purpose of this design was not to infer strict causality from a perfectly orthogonal experiment, but to identify which variables and compound classes deserved the highest priority for subsequent controlled testing. This distinction is crucial, because several variables co-varied through recipe logic and process-control architecture, especially around throughput and adhesive dosing.

Accordingly, the statistical language throughout the manuscript has been restricted to “process–state contrasts”, “directional responses”, and “operational associations”. This terminology is used intentionally: in a commercial MDF line, several variables are coupled by mass balance, recipe logic, energy demand, and control loops. The purpose of the analysis is therefore to reveal chemically interpretable control domains and high-priority hypotheses for confirmatory experimentation, not to present line-independent emission factors or isolated mechanistic coefficients.

The adhesive system was treated as a mechanistically important source term because aminoplastic resin chemistry can contribute to formaldehyde and other oxygenated compounds during blowline application, drying, curing, and hydrolytic stress. During the analysed campaign, the plant used commercial low–formaldehyde amino resin adhesives from the UF/MUF adhesive portfolio used for MDF/HDF manufacture. Production metadata identified UF-type DL-series grades and a MUF-type Me-series grade within the campaign-relevant adhesive system, with no resin formulation, resin batch, or adhesive recipe change that would affect the evaluated process–state contrasts. Supplier documentation for the relevant UF/MUF adhesive families describes aqueous urea–formaldehyde or melamine–urea–formaldehyde condensation products with solids contents typically around 63–66%, pH values around 7.8–9.2, densities around 1.27–1.30 g cm^−3^ at 20 °C, and fresh-resin free formaldehyde below 0.1%. The exact grade-by-grade assignments to product recipes, detailed molar ratios, and recipe-specific formulation variables are commercially sensitive and are therefore not disclosed.

For the transparency of the industrial dataset, the measurement campaign was defined as a discontinuous Q1 2022 industrial campaign covering the windows 5–27 January 2022, 15–16 February 2022, 24–25 February 2022, and 1–4 March 2022. The FTIR raw-data workbook contained 85,031 valid time-stamped concentration records acquired at intervals of approximately 22 s, whereas the process–state analysis used the subset of emission and plant-operating records that could be synchronized with stable production annotations. Start-up, shutdown, cleaning, maintenance, measurement failure, and product transition periods were excluded when they were identifiable from the plant records. Because each A/B contrast was defined separately for a specific process variable, the retained group sizes differed among contrasts; this imbalance is reported together with the corresponding process-state contrasts as the nB/nA ratio and is the reason why the analysis is interpreted as process–state screening rather than as a balanced factorial design. Non-confidential production metadata indicate that the campaign covered HDFFUB and HDFFUN fibreboard product families with nominal thicknesses from 5.7 to 7.7 mm. Exact recipes, densities, product-assignable setpoints, and resin-grade mapping data are commercially sensitive and are therefore not disclosed.

### 2.2. Analytical Measurements and Response Variables

TVOC at the humid and hot stationary source was evaluated as a propane-equivalent total-carbon/FID signal within the industrial emission-measurement campaign documented by DIEFFENBACHER GmbH Maschinen- und Anlagenbau (Eppingen, Germany). Compound-resolved analysis was performed by Fourier-transform infrared (FTIR) spectroscopy using a Gasmet DX4000 analyser operated with the manufacturer’s FTIR analysis software (Gasmet Technologies Oy, Vantaa, Finland) and a 5.0 m optical path length [11,31,32,33]. The combined use of FID-type total-carbon tracking and FTIR speciation was methodologically advantageous: the former provided a robust total-carbon indicator for plant-scale comparison, while the latter resolved the chemically distinct compounds needed to interpret the origin of the emission signal and its process sensitivity.

Analytes were selected from literature evidence, preliminary plant analyses, and empirical process knowledge. For the compositional analysis, the relative contribution of compound i to the resolved VOC sum was calculated as p_i = c_i/Σc_j × 100%. To facilitate comparison among heterogeneous process variables while protecting industrial confidentiality, graphical relationships were expressed in normalized units referenced to the maximum observed value within each variable. The interpretation of these normalized trends therefore focused on relative directionality, response magnitude, and clustering structure rather than on disclosure of absolute setpoints.

To improve analytical traceability, the available Q1 2022 FTIR concentration data are distinguished from proprietary spectral-processing information. The Q1 2022 dataset contains the time-stamped FTIR concentration outputs used for the compound-resolved analysis, and the target analyte set is documented in the Methods and Supplementary Tables S1 and S2. However, the original Q1 2022 spectra, proprietary FTIR spectral libraries, and reference-spectrum fitting outputs are not available to the authors in a form suitable for public disclosure. Later plant measurements include illustrative spectra and fit checks, but these were not used as direct evidence for the Q1 2022 dataset and are therefore not presented as original Q1 supporting spectra. The article and supplementary data-description package therefore provide the maximum publishable analytical transparency—instrument type, sampling location, target analytes, temporal resolution, raw concentration records, and dataset/filtering metadata—while explicitly avoiding disclosure of proprietary FTIR-library and absolute-emission information. Calculation of relative contributions, normalized process-state contrasts, response summaries, and exploratory decorrelation/principal-component analysis was performed in R software, version 4.5.3 (R Foundation for Statistical Computing, Vienna, Austria).

### 2.3. Screened Process Variables

Ten process variables were screened because they were expected, on mechanistic grounds, to influence either VOC generation, VOC transport, or gas-cleaning efficiency: hardwood share in the furnish, digester residence time, discharge screw speed, adhesive amount, urea amount, dryer inlet temperature, dryer fan power, additional flue-gas supply, scrubber–water pH, and scrubber–water temperature. Together, these variables span the three major physicochemical domains relevant to MDF dryer emissions: (i) raw material chemistry, (ii) thermo-reactive loading of the fibre/resin system, and (iii) conditioning of the gas stream during downstream wet cleaning. The screened variables and their expected emission relevance are summarized in Table 1.

### 2.4. Selection of Representative Compounds and Exploratory Multivariate Analysis

The initial compositional evaluation showed that α-pinene, 3-carene, limonene, formaldehyde, and methanol dominated the resolved VOC signal. Because α-pinene and 3-carene were strongly correlated, and because strong empirical relationships were also observed between limonene and propane and between formaldehyde and acetaldehyde, α-pinene, limonene, methanol, and formaldehyde were retained as representative compounds for the comparative screening. To explore the coupled structure of the process variables, an additional decorrelation/principal component step was used as an exploratory dimensionality-reduction tool. This step was intended to identify dominant operational domains, not to replace mechanistic interpretation.

### 2.5. Data Handling, Seasonal Context, and Interpretation of Process–State Effects

Because the dataset was acquired from a commercial production line, the analysis was designed as a process–state screening and not as an all-season emission factor study. The Q1 2022 period represented regular plant operation with representative production line KPIs and stable line speed, while the binder composition remained unchanged in a way relevant to the present contrasts. Incoming wood chips before thermomechanical pulping typically contained approximately 25–35% moisture, and the plant weather file for the actual measurement windows showed ambient temperatures from approximately −4.3 to 10.0 °C and relative humidity from approximately 36.5 to 93.9%. Later internal plant measurements in 2024 showed qualitatively repetitive directional behaviour for the relevant emission domains, including terpene behaviour, but these measurements were not an orthogonal multi-season validation of the Q1 2022 dataset. The observed responses are therefore reported as directional associations under the campaign conditions and should be used to prioritize causal experiments rather than to claim universal transferability across all seasons, furnish stocks, and product recipes.

Special caution was applied to process variables that can be affected by hidden co-variation, particularly scrubber–water temperature. A plant correlation check indicated that scrubber–water temperature co-varied with discharge screw speed/fibre throughput (r approximately 0.66) and dryer inlet temperature (r approximately 0.77), whereas no dominant independent effect of flue-gas supply or product type could be isolated from the available process records. The temperature was independently checked by handheld measurement, but under full-scale operation, it still reflected the coupled source-generation and abatement state of the line. Its effect is therefore described as an operational association involving gas–liquid partitioning, thermal load, and abatement conditions, not as an isolated causal coefficient.

## 3. Results

### 3.1. Dominant Compounds in the Cleaned Dryer Exhaust

**Figure 1:**
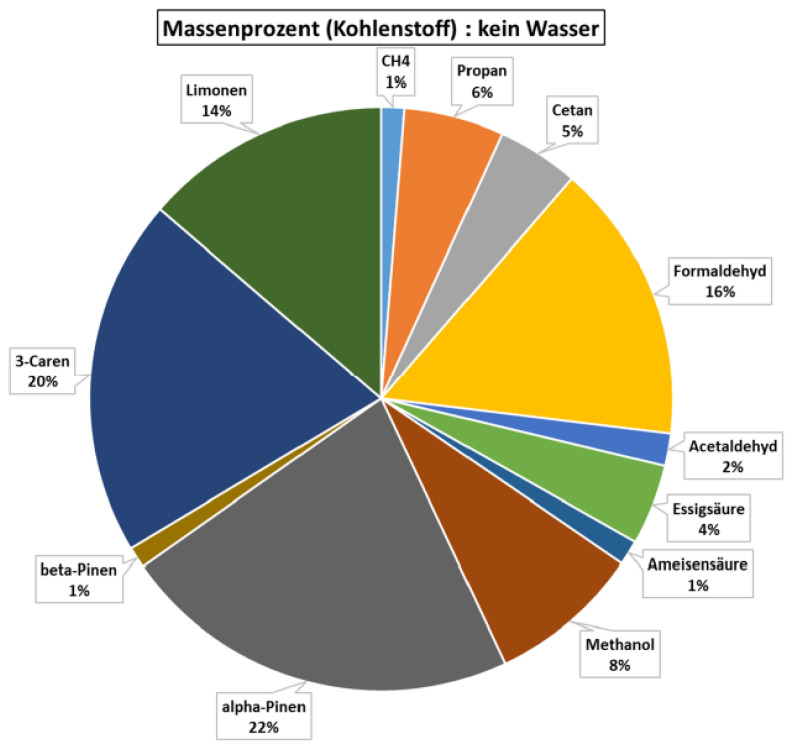
Relative contribution of the dominant FTIR-resolved compounds to the cleaned dryer-stack VOC signal.

The compositional screening demonstrated a pronounced hierarchy in the cleaned stack signal: α-pinene, 3-carene, limonene, formaldehyde, and methanol together accounted for more than 80% of the resolved VOC mixture. The relative contributions of the dominant compounds are shown in Figure 1. This result shows that, despite the chemical complexity of a full-scale MDF line, the final post-abatement stack signature is not distributed randomly across many weak analytes but concentrated in a limited chemotype that combines terpene markers of wood extractives with oxygenated volatiles and adhesive-sensitive carbonyl chemistry. In practical terms, the cleaned exhaust remained sufficiently structured to permit compound-level interpretation rather than only bulk TVOC tracking. The dominance of this five-compound group also justifies the use of a reduced marker set for subsequent process–state screening, because most of the resolved emission burden was captured by compounds with chemically interpretable origins.

**Figure 2:**
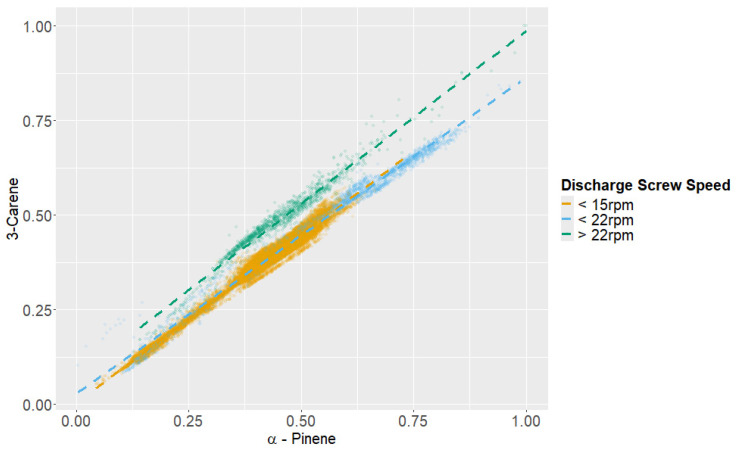
Relationship between α-pinene and 3-carene across the full dataset (normalized units). Shaded bands represent the uncertainty bands of the fitted trend lines.

Within this dominant fraction, α-pinene and 3-carene showed a strong positive relationship across the plant dataset, supporting the interpretation that both compounds are governed by the same wood-derived source domain and respond coherently to furnish and process changes. The corresponding relationship is shown in Figure 2. The observed covariance indicates that, at the level of industrial screening, the terpene signal contained substantial redundancy. This justified the use of α-pinene as a representative terpene marker in the subsequent process–state analysis and allowed the later comparisons to focus on orthogonality between terpene-, alcohol-, and carbonyl-dominated response behaviour rather than on parallel tracking of highly collinear monoterpenes.

### 3.2. Process–State Contrasts for Representative Compounds

Table 2 summarizes the relative change of the representative compounds when the operating state was shifted from A (lower setting) to B (higher setting) for each screened variable. A robust directional pattern emerged. Variables related to hydrothermal severity, fibre throughput, adhesive/urea dosing, dryer thermal intensity, and scrubber–water temperature predominantly increased the representative compounds, whereas hardwood-rich furnish, additional flue-gas supply, and more alkaline scrubber–water decreased them. The dataset therefore separates the screened variables into two broad functional groups: source strength variables that intensify compound liberation, formation, or transfer into the gas phase, and conditioning variables that dilute, absorb, or otherwise attenuate the final cleaned stack signature. The magnitude of the responses also shows that the same process variable can affect the four markers very differently, which argues against using a single compound or only TVOC as a universal proxy for all emission state changes. Importantly, the contrast matrix was built from normalized plant data acquired under industrial operating variability; the table should therefore be read as a map of relative operational sensitivity rather than as a set of transferable absolute emission factors.

Among the positive responses, several contrasts were especially informative. Increasing digester residence time increased methanol by 106%, limonene by 85%, and α-pinene by 72%, indicating that the severity of upstream hydrothermal treatment measurably propagated into the final cleaned stack chemistry. Increasing dryer inlet temperature raised formaldehyde by 87%, limonene by 83%, and methanol by 67%, supporting a thermally intensified emission regime in which both wood-derived and adhesive-related components respond strongly to heat input. Circulating water temperature in the scrubber produced similarly strong increases, especially for limonene (90%) and formaldehyde (72%), demonstrating that the post-abatement stack signal was controlled not only by primary generation within the fibre–resin system but also by downstream partitioning and removal efficiency. In contrast, dryer fan power produced almost no net change, suggesting that, within the studied operating range, the dominant control mechanism was not the gross gas transport alone but rather the coupled chemistry of source formation and gas conditioning. The screened process states therefore reveal a chemically structured system in which upstream source terms and downstream abatement terms remain simultaneously visible in the final stack response.

The principal result of this contrast matrix is therefore not the individual percentage change alone, but the separation of the cleaned stack signal into interpretable source, transport, and abatement sensitivities. Hydrothermal severity and dryer temperature behaved as thermochemical drivers; discharge screw speed and adhesive/urea dosing behaved as throughput-recipe drivers; and circulating water temperature and pH represented downstream abatement-state drivers. This separation gives the dataset practical value beyond literature confirmation: it converts a complex VOC mixture into a ranked set of production levers that can be tested in a subsequent controlled campaign.

**Figure 3:**
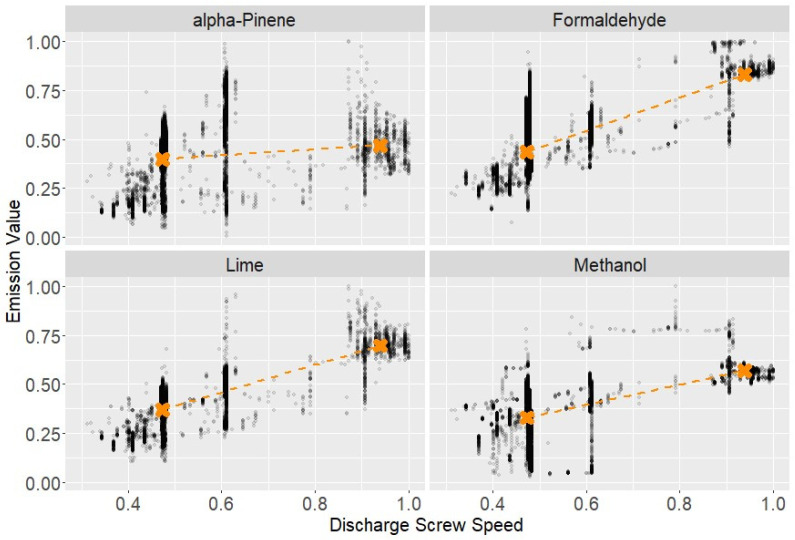
Effect of discharge screw speed on representative compounds (normalized units). Orange markers indicate cluster centroids of the contrasted process states. Black points represent individual normalized observations; orange markers indicate cluster centroids and the dashed orange line connects the centroids.

**Figure 4:**
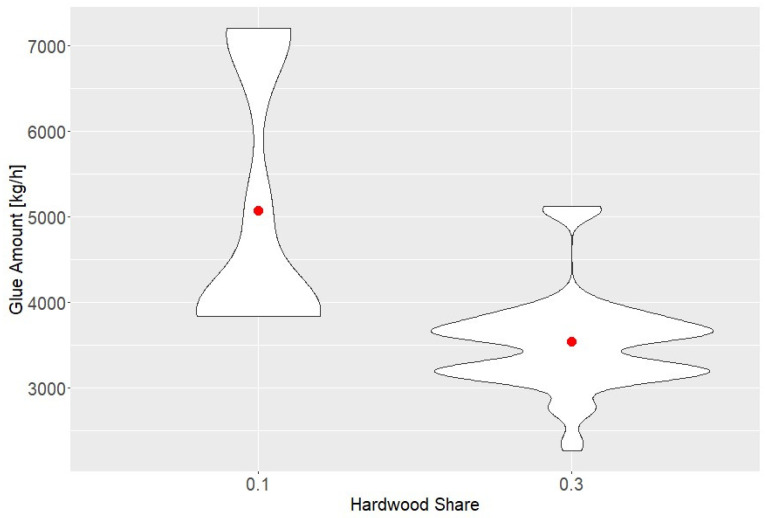
Relationship between hardwood share and adhesive amount (normalized units). The red points indicate group means.

**Figure 5:**
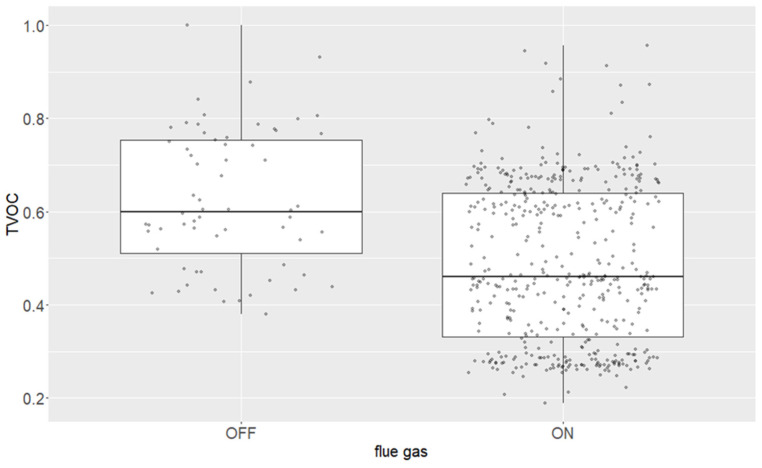
TVOC response to additional flue-gas supply (normalized units). Dots represent individual normalized observations.

**Figure 6:**
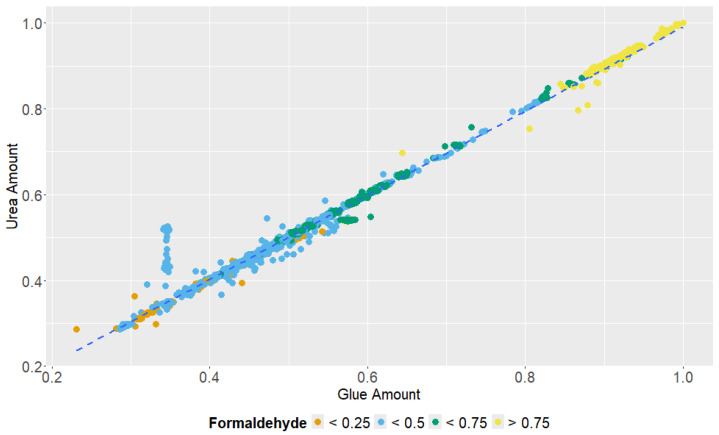
Relationship between adhesive amount and urea amount (normalized units). Colour scale indicates normalized formaldehyde level. The dashed line indicates the fitted trend.

The corresponding normalized relationships are illustrated for discharge screw speed (Figure 3), hardwood share versus adhesive amount (Figure 4), additional flue-gas supply (Figure 5), and adhesive amount versus urea amount/formaldehyde response (Figure 6).

### 3.3. Differential Responsiveness of the Representative Compounds

The four representative compounds did not respond equally to changes in the process state. α-Pinene exhibited the most damped overall response span, whereas limonene, methanol, and formaldehyde reacted much more strongly to the screened process shifts. This result means that dominance in composition does not automatically imply diagnostic sensitivity. A compound may represent a large fraction of the emission burden and yet behave comparatively conservatively with respect to routine operating changes. From a process-monitoring perspective, this separation between abundance and responsiveness is important because it identifies limonene, methanol, and formaldehyde as higher-gain indicators of process–state disturbance, while α-pinene functions more as a stable marker of the wood-derived terpene background. The result also anticipates the discussion that follows: industrial monitoring should prioritize compounds not only by abundance, but by mechanistic interpretability and response amplitude. The mean negative and positive response magnitudes are summarized in Table 3.

### 3.4. Exploratory Grouping of Correlated Process Variables

**Figure 7:**
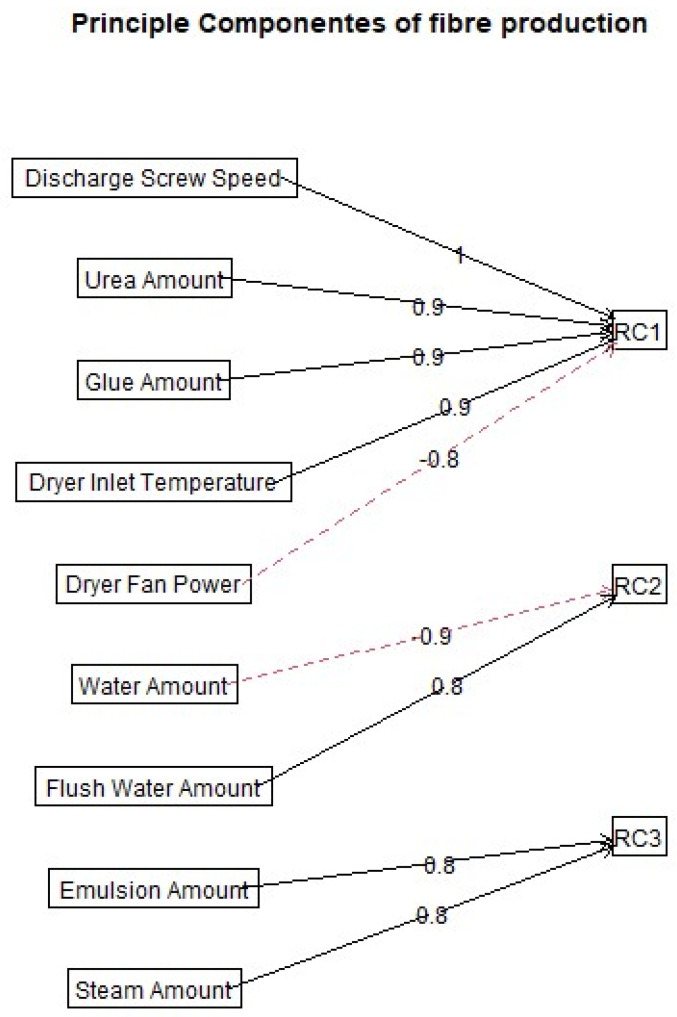
Exploratory grouping of process variables into three principal operational components (RC1–RC3). Solid lines indicate positive loadings and dashed lines indicate negative loadings.

The exploratory decorrelation/principal component step showed that the observed process interactions could be reduced to a small number of operational domains. RC1, which grouped the drying- and throughput-related variables, explained about 50% of the variance in the screened process space. RC2 and RC3 together explained roughly 35% and represented material- and water-related domains. The importance of this result lies not only in data reduction but in process interpretation: the industrial emission problem was shown to be organized around a limited number of coupled domains rather than around ten independent levers. Such structure is precisely what is needed before designing causal factorial experiments, because it indicates where orthogonalization efforts should be concentrated and where strong co-variation is likely to confound naïve single-factor interpretation. In other words, the multivariate grouping converts a descriptive screening campaign into a strategically useful experimental map. The resulting exploratory grouping is shown in Figure 7.

## 4. Discussion

### 4.1. Scientific Meaning of the Dominant Emission Profile

The dominance of α-pinene, 3-carene, limonene, methanol, and formaldehyde is chemically coherent and consistent with the broader literature on wood and wood-based panels, which repeatedly identifies terpenes, carbonyls, alcohols, and related oxygenated compounds as the core VOC families in MDF-related systems [6,7,13,15,20,29,30,34]. Adamová et al. summarized monoterpenes and carbonyl compounds as recurrent major contributors in wood and panel emissions [7], while Gonçalves et al. recently showed that dry-process fibreboard releases a structured carbonyl fraction including formaldehyde, acetaldehyde, and higher aldehydes rather than an undifferentiated VOC background [20]. He et al. demonstrated that the species composition changes markedly between wood chip, resin-coated fibre, and finished panel [13]. Our stack data extend these observations to the process scale: even after industrial cleaning, the emission signature collapses into a chemically interpretable marker set rather than a diffuse residual carbon signal.

At the same time, direct numerical comparison with most published values must be performed cautiously. Many reference studies report chamber concentrations, emission factors from finished boards, or emissions from untreated process stages, whereas the present dataset describes relative compound responses in the cleaned stack after wet scrubbing and biological wastewater treatment. The scientific comparison is therefore qualitative and mechanistic rather than absolute. What can be compared robustly is the rank order of dominant compound classes, the direction of response to process perturbation, and the persistence of source information after abatement. On that basis, the present results are fully consistent with the literature while adding a missing scale of observation: the real, abated industrial exhaust.

This distinction is central to the novelty of the study. Product–emission studies answer how a panel behaves under standardized or simulated use conditions; manufacturing-stage studies answer how individual materials or intermediate products emit before the full industrial chain is complete. The present cleaned stack dataset answers a different question: which chemical information survives the entire sequence of fibre preparation, aminoplastic resin addition, drying, particulate separation, wet scrubbing, and biological post-treatment, and remains visible at the regulated emission point? The persistence of a structured marker set after this chain is the key scientific observation.

### 4.2. Mechanistic Interpretation of the Screened Variables

The effect of hardwood share is one of the most interpretable results and is in agreement with MDF-specific species studies. In general, coniferous furnish has a higher monoterpene load, whereas hardwood-rich furnish tends to suppress terpene emissions and shift the oxygenated profile [9,15]. Gabriel et al. reported that lowering the hardwood fraction in MDF increased terpenes and aliphatic aldehydes [9], and Jabbari et al. likewise showed that species choice strongly controls VOC intensity and profile in commercial panels [21]. The hardwood furnish in the present plant—oak (*Quercus* spp.), beech (*Fagus* spp.), birch (*Betula* spp.), and poplar (*Populus* spp.)—is therefore expected to reduce wood-derived terpene loading relative to softwood-rich furnish. The present dataset, however, also reveals why industrial interpretation must go beyond species chemistry alone: higher hardwood share coincided with lower adhesive demand. The observed reductions in methanol and formaldehyde are thus best interpreted as a combined furnish-composition and recipe-response effect, not as a purely botanical effect. This distinction matters because it shows that industrial furnish changes can propagate to emissions indirectly through formulation logic.

The strong positive responses to digester residence time and dryer inlet temperature support a severity-based interpretation of VOC generation. Longer hydrothermal pretreatment increases the time available for deacetylation, extractive release, cleavage of methylated wood structures, and formation of oxygenated volatiles, while higher dryer temperatures intensify volatilization and thermally promoted conversion reactions [13,14,16,22,23,24]. The wood-drying literature has long shown that emissions can rise sharply when thermal load increases or when moisture content approaches critical ranges during drying [22,23,24]. MDF-specific chemistry studies point in the same direction. Roffael and co-workers showed that pulping route and pretreatment alter the balance between formaldehyde and volatile organic acids in fibres and MDF [14], and He et al. showed that process stages reshape both the species distribution and the origin of emitted compounds [13]. From the environmental-control side, Liang et al. demonstrated in a full-scale experimental room and in controlled chamber studies that temperature and humidity strongly affect formaldehyde emission behaviour from MDF [25,26,27,28]. Our results complement these product- and material-oriented studies by showing that hydrothermal severity and thermal load remain visible even at the level of the final cleaned industrial stack.

Discharge screw speed behaved as a master operational variable because it governs much more than fibre transport. Raising screw speed increases fibre throughput, elevates the mass of material entering drying, increases the demand for adhesive and urea addition, and forces the system toward higher thermal duty to maintain target outlet conditions. This integrated response explains why discharge screw speed produced clear positive shifts in the representative compounds. A similar logic applies to adhesive and urea amount. He et al. showed that formaldehyde in wood-based panels is dominated largely by UF-type resin rather than by wood chips [13], and Kim et al. demonstrated that resin content strongly influences formaldehyde release in MDF pressing experiments even when its effect on TVOC can be weaker than that of temperature [10]. Product-side work from Antov et al. further showed that reducing UF content and reformulating the binder system can drive board formaldehyde content toward near-natural-wood levels [17]. The present industrial data add the process-scale complement to these findings: under commercial operation, resin dose and urea dose are not only formulation variables but also high-leverage emission state variables.

From a polymer chemistry perspective, this result is particularly relevant for a journal focused on polymeric materials. UF and MUF adhesives are not inert process additives: they are reactive aminoplastic networks, the final structure of which depends on resin formulation, molar ratio, curing environment, moisture, pH, and thermal history. Under blowline and dryer conditions, residual-free formaldehyde, reversible methylol and methylene-ether chemistry, hydrolysis of incompletely cured structures, and scavenger reactions can all contribute to the oxygenated VOC signature. The observed sensitivity of formaldehyde to adhesive amount, urea amount, and dryer inlet temperature is therefore consistent with a coupled wood–polymer/resin chemistry origin rather than with a purely botanical emission source.

Additionally, flue-gas supply, scrubber–water temperature, and scrubber–water pH illustrate that end-of-pipe conditioning is not a passive background factor but part of the emission system itself. In the screened operating states, additional flue-gas supply lowered TVOC and the representative compounds, whereas warmer scrubber–water increased them and higher pH slightly decreased them. At least three mechanisms are plausible and not mutually exclusive: dilution by higher gas volume, altered effective drying intensity upstream, and compound-selective absorption or transformation in the scrubber/biological treatment train. Reviews of wood panel VOC control emphasize that abatement performance depends on compound class, treatment principle, mass-transfer characteristics, and economic constraints [29,30]. That framework fits the present data well because terpenes, methanol, and formaldehyde differ substantially in water solubility, reactivity, and biodegradability. The weak effect of dryer fan power, by contrast, suggests that within the observed operating range bulk airflow alone was less decisive than chemistry and phase partitioning. In practical terms, the data argue that industrial VOC mitigation must treat source generation and gas cleaning as a coupled control problem.

### 4.3. Differential Compound Sensitivity and Implications for Monitoring

The unequal responsiveness of the marker compounds is scientifically useful. α-Pinene represented a major fraction of the stack profile yet showed the smallest average response span, whereas limonene, methanol, and formaldehyde responded much more strongly to process–state shifts. This separation between abundance and responsiveness is important because it means that the largest compound is not automatically the best process indicator. α-Pinene behaves mainly as a background tracer of softwood/extractive loading, while limonene appears more sensitive to operational perturbation within the terpene class. Methanol and formaldehyde act as higher-gain markers of hydrothermal severity, thermal load, and adhesive-related chemistry. This hierarchy is consistent with prior mechanistic understanding: native extractive markers are buffered by furnish inventory, whereas methanol and formaldehyde are strongly influenced by reactive conversion pathways and amino resin chemistry [6,7,8,12,13,16,27,28].

The analytical implication is that advanced industrial monitoring should combine a total-carbon indicator with a targeted compound panel rather than relying on TVOC alone. The recent methodological review by Perera et al. makes the same point in broader engineered wood terms: method choice determines which scientific question can be answered, and process diagnosis benefits from fast or online compound-resolved strategies [11]. The present results suggest a practical marker set for MDF dryer control: α-pinene or 3-carene for wood-derived extractive loading, limonene for responsive terpene-class behaviour, methanol for hydrothermal wood conversion, and formaldehyde for amino-resin-related and thermally intensified oxygenated chemistry. Cross-checking such a marker set with periodic TD-GC/MS or PTR-MS campaigns would provide both operational speed and analytical specificity [11,20,21].

### 4.4. Practical Guidance for Industrial Monitoring and Mitigation Design

The value of the present dataset is not that it repeats the well-established observation that wood-based panels emit terpenes, alcohols, carbonyls, and organic acids. Its value is that it identifies which of these chemical families remain process-diagnostic at the final cleaned stack after the complete industrial chain of defibration, blowline resin addition, flash-drying, dust separation, wet scrubbing, and biological wastewater treatment. This changes the interpretation from descriptive emission profiling to operational decision support.

Three practical outputs follow. First, routine monitoring should combine TVOC with a deliberately small marker panel: α-pinene or 3-carene for wood-extractive loading, limonene for a more process-sensitive terpene response, methanol for hydrothermal wood conversion, and formaldehyde for aminoplastic resin chemistry and thermal stress. This panel would provide more diagnostic resolution than TVOC alone while remaining realistic for industrial use.

Second, mitigation experiments should be prioritized according to the ranked operational domains identified here. The highest-priority domain is the coupled drying/throughput/resin domain, especially digester residence time, discharge screw speed, adhesive amount, urea amount, and dryer inlet temperature. These variables jointly control source generation and mass loading and should therefore be orthogonalized in the next designed campaign. Dryer fan power, by contrast, was a lower-priority lever within the studied operating range.

Third, candidate attenuation and abatement-state levers—higher hardwood share, additional flue-gas supply, scrubber–water pH, and the scrubber–water temperature response—should be evaluated under controlled conditions to separate true mitigation from dilution, recipe logic, or correlated operating states. In this sense, the study does not merely describe a plant-specific dataset; it provides a ranked experimental roadmap for developing lower-emission MDF dryer operation while preserving industrial feasibility.

### 4.5. Limitations and Future Work

The major limitation of the present study is the observational character of the dataset. The production line was operated under commercial conditions, not under an orthogonal experimental matrix, and several contrasts were imbalanced or structurally coupled through recipe logic and control architecture. In addition, the measured signal was the cleaned stack after scrubbing and biological treatment, so upstream generation, gas-phase transport, liquid-phase absorption, and biological removal could not be experimentally disentangled within one campaign. For that reason, the reported magnitudes should be interpreted as robust industrial effect sizes for prioritization, not as pure causal coefficients or universally transferable emission factors. Yet this limitation is inseparable from the main strength of the study: the data capture the real coupled complexity that laboratory boards and chamber studies necessarily simplify.

Seasonal effects require particular caution. The analysed campaign was conducted under winter-to-early-spring Q1 conditions, with incoming wood-chip moisture typically around 25–35% before thermomechanical pulping and ambient temperatures during the actual measurement windows ranging from approximately −4.3 to 10.0 °C. The period reflected regular line operation and no relevant seasonal change of binder composition was present; however, operating modes can still adapt marginally throughout the year in response to wood temperature, wood moisture, ambient conditions, and raw material properties. Subsequent internal plant measurements indicated qualitatively repetitive directional behaviour, but a formally controlled multi-season validation was not part of the present dataset. The study therefore treats multi-season confirmation as a necessary next step rather than as an already completed validation.

The scrubber–water temperature response should also be interpreted conservatively. Warmer circulating water can reduce absorption efficiency for soluble or semi-soluble compounds and can affect phase partitioning; however, the available plant correlation check showed that this variable co-varied with throughput-related and dryer temperature variables. Consequently, scrubber–water temperature is not interpreted as a fully isolated control lever. Instead, it is treated as a high-priority operational marker for the coupled source-generation/abatement state and as a candidate variable for a deliberately controlled follow-up experiment.

The next research step should therefore be a designed industrial campaign rather than a broader observational one. A factorial or D-optimal programme should focus first on the dominant RC1 variables—especially discharge screw speed, adhesive dose, urea dose, digester severity, and dryer inlet temperature—while controlling furnish and recipe confounders as tightly as commercial operation permits. In parallel, stage-wise mass balancing should be introduced so that digester condensate or steam, blowline aerosol, dryer inlet, dryer outlet, scrubber liquor, and final stack are sampled within the same campaigns. Only that type of coordinated design can separate source formation, partitioning, and abatement. Finally, the FTIR/FID framework should be cross-validated against orthogonal methods such as TD-GC/MS or PTR-MS for selected transient states and marker compounds [11,20,21]. Such a programme would move the field from industrial screening toward predictive, mitigation-oriented process science.

This interpretation also makes the limits of transferability explicit. The relative trends are intended for ranking and hypothesis generation under the investigated industrial conditions. Absolute emission factors, all-season stability, and independently controlled abatement effects require deliberately designed validation campaigns with extended operational metadata and, where industrial confidentiality permits, stage-wise mass-balance information.

## 5. Conclusions

This study provides compound-resolved evidence from a full-scale industrial MDF dryer and shows that the cleaned stack signature is concentrated in a small number of chemically meaningful marker compounds. α-Pinene, 3-carene, limonene, methanol, and formaldehyde together represented more than 80% of the resolved VOC mixture, demonstrating that even a complex industrial emission profile can be interpreted through a limited chemotype.

The most influential process–state contrasts were associated with hydrothermal severity, throughput-linked loading, adhesive/urea dosing, and thermal operating intensity. Higher digester residence time, discharge screw speed, adhesive amount, urea amount, dryer inlet temperature, and scrubber–water temperature increased one or more representative compounds, whereas higher hardwood share, additional flue-gas supply, and higher scrubber–water pH decreased them. Limonene, methanol, and formaldehyde were the most sensitive reporters of these changes.

For industrial implementation, the results indicate that MDF dryer emission control can be approached through a reduced set of chemically meaningful markers and coupled operational domains, rather than through undifferentiated TVOC values alone. The work therefore provides a defensible scientific basis for moving from compliance monitoring toward targeted, experimentally testable emission-mitigation strategies in full-scale MDF manufacture.

## Figures and Tables

**Table 1:** Screened process variables and their expected relevance to dryer-stack VOC composition.

Process Variable	Operational Interpretation	Expected Link to VOC Emissions
Hardwood share	Furnish composition	Changes the terpene burden of the incoming wood and may also co-vary with adhesive demand.
Digester residence time	Severity of thermal pretreatment/defibration	Affects liberation and formation of extractives, methanol, and carbonyl compounds before drying.
Discharge screw speed	Master throughput variable	Changes fibre mass flow and co-varies with downstream additive dosing and gas loading.
Adhesive amount	UF/MUF resin input	Increases the availability of adhesive-related formaldehyde and resin-derived VOCs.
Urea amount	Scavenger/recipe variable	Reflects formaldehyde-control chemistry but also co-varies with adhesive amount and production recipe.
Dryer inlet temperature	Thermal load of the dryer	Controls volatilization and thermally induced release of wood- and adhesive-derived compounds.
Dryer fan power	Gas transport and residence-time control	May influence dilution, transport, and stack concentration, although not necessarily VOC generation.
Flue-gas supply	Supplementary hot-gas input from the energy plant	Can alter dilution, oxidation potential, and the effective dryer inlet temperature.
Circulating water pH	Scrubber chemistry	May modify the absorption/removal of polar compounds during wet cleaning.
Circulating water temperature	Scrubber operating condition	Affects gas–liquid partitioning and the removal efficiency of soluble species.

**Table 2:** Relative change in representative compounds after shifting from process state A to process state B (higher parameter level).

Process Variable	α-Pinene	Limonene	Methanol	Formaldehyde	nB/nA
Hardwood share	−17%	−23%	−21%	−31%	2.76
Digester residence time	72%	85%	106%	18%	0.78
Discharge screw speed	31%	45%	42%	47%	0.16
Adhesive amount	14%	71%	38%	54%	0.82
Urea amount	9%	81%	70%	83%	0.05
Dryer inlet temperature	11%	83%	67%	87%	0.05
Dryer fan power	0%	0%	2%	1%	0.92
Flue-gas supply	−15%	−32%	−41%	−27%	7.31
Circulating water pH	−7%	−11%	−28%	−19%	1.26
Circulating water temperature	48%	90%	77%	72%	0.05

**Table 3:** Mean response magnitudes of the representative compounds across all screened parameter contrasts.

Representative Compound	Mean Negative Response	Mean Positive Response	Response Span
α-Pinene	−13%	31%	44%
Limonene	−22%	76%	97%
Methanol	−30%	67%	97%
Formaldehyde	−26%	60%	86%

## Data Availability

Aggregated results supporting the conclusions are included in the article and the supplementary data-description tables. The underlying raw plant-control datasets, detailed recipe assignments, proprietary FTIR library/fitting outputs, and absolute industrial emission values are not publicly available because they contain commercially sensitive information from SWISS KRONO TEX GmbH & Co. KG. Access to additional aggregated information may be considered by the corresponding author subject to approval by SWISS KRONO TEX GmbH & Co. KG.

## Supplementary Materials

The following supporting information can be downloaded at: https://www.mdpi.com/article/10.3390/polym18101230/s1, A supplementary data description package supports the compound-resolved interpretation: Table S1: FTIR/FID target-analyte and marker-compound information used for the screening; and Table S2: Dataset-window, operating-mode, resin-system, and confidentiality metadata. Original Q1 2022 spectra, proprietary FTIR spectral library/fitting outputs, detailed recipe mapping, and absolute emission values are not publicly disclosed because they are industrially sensitive data generated in an industry-linked PhD research collaboration with SWISS KRONO TEX GmbH & Co. KG.
