# Supplementary material for: Compound-Resolved VOC Dynamics in a Full-Scale Medium-Density Fibreboard Dryer: Process–State Screening Across Wood Furnish, Amino Resin Dosing, and Thermal Operating Variables
Source: Polymers (Basel). 2026 May 18;18(10):1230. doi: 10.3390/polym18101230 (PMC13210732; doi:10.3390/polym18101230)
Supplement: Supplementary file 1 [file polymers-18-01230-s001.zip › polymers-4285972-supplementary.pdf]

Supplementary Data-Description Tables

Manuscript ID: polymers-4285972

Manuscript title: Compound-Resolved VOC Dynamics in a Full-Scale Medium-Density Fibreboard Dryer: Process-State Screening across Wood Furnish, Amino-Resin Dosing, and Thermal Operating Variables

This supplementary file provides the maximum publishable analytical and dataset-level traceability for the article. Original Q1 2022 FTIR spectra, proprietary spectral-library/fitting outputs, detailed recipe-level resin mapping, and absolute industrial emission values are not publicly disclosed because they are commercially sensitive industrial data generated in an industry-linked PhD research collaboration with SWISS KRONO TEX GmbH & Co. KG.

Table S1. FTIR/FID target-analyte and marker-compound information used for the screening.

Analyte or signal	Analytical role in the study	Interpretive domain
TVOC mgC (FID, propane-equivalent)	Bulk total-carbon-type indicator used for plant-scale comparison and regulatory-style tracking.	Overall VOC burden / compliance-type signal
H2O, CO2, CO, N2O, NO, NO2, NH3	FTIR-resolved gas-matrix and combustion/process-related components retained as supporting concentration outputs.	Gas matrix / combustion and process context
Methane, ethane, propane, long-chain hydrocarbons	Hydrocarbon background signals; propane was also used as a comparison signal in empirical relationship checks.	Hydrocarbon background and gas-phase transport context
Formaldehyde	Dominant carbonyl marker and principal amino-resin-sensitive oxygenated compound.	UF/MUF resin chemistry, residual/free formaldehyde, hydrolysis, thermal stress
Acetaldehyde	Secondary carbonyl used in relationship checks with formaldehyde.	Wood/resin-related carbonyl chemistry
Acetic acid, formic acid	Organic acid outputs included in the FTIR concentration dataset and source-chemistry context.	Thermo-hydrolytic/oxidative wood chemistry
Methanol	Representative alcohol marker retained for process-state screening.	Hemicellulose deacetylation and hydrothermal wood conversion
alpha-Pinene, beta-pinene, d3-carene, limonene	Monoterpene markers of wood extractives; alpha-	Furnish/extractive inventory and thermally assisted

	pinene, 3-carene, and limonene defined the terpene-dominated response domain.	volatilization

Table S2. Dataset-window, operating-mode, resin-system, and confidentiality metadata.

Metadata item	Publishable information
Industrial site and system	Full-scale MDF production line at SWISS KRONO TEX GmbH & Co. KG, Heiligengrabe, Germany; single-stage flash-tube fibre dryer followed by wet scrubbing and biological wastewater post-treatment. Measurements were performed on the clean-gas side of the stack.
Measurement windows	Discontinuous Q1 2022 campaign: 5-27 January 2022, 15-16 February 2022, 24-25 February 2022, and 1-4 March 2022.
FTIR concentration records	85,031 valid time-stamped FTIR concentration records were available in the Q1 2022 raw-data workbook. The acquisition interval was approximately 22 s.
Plant records and process-state screening	More than 20,000 synchronized plant-operating records were used for the process-state screening after matching emission and operating data with stable production annotations.
Operating-mode filtering	Start-up, shutdown, cleaning, maintenance, measurement-failure, and product-transition periods were excluded where identifiable from plant records. A/B contrasts were defined separately by screened process variable, resulting in different nB/nA ratios.
Product and recipe disclosure boundary	The disclosed plant metadata identify the product families HDFFUB and HDFFUN and nominal board thicknesses between 5.7 and 7.7 mm. Detailed density classes, recipe mapping, product-assignable resin grades, operating setpoints, and absolute emission values are industrially confidential.
Resin system	Production metadata identified a campaign dominated by UF-type KronoGlue DL-series adhesive grades, principally DL7109, DL7112, and DL7161, used for MDF/HDF production. MUF grades were part of the plant adhesive portfolio, but detailed grade-by-recipe mapping is commercially sensitive. The fresh-resin free formaldehyde content was below 0.1%; exact molar ratios and recipe-specific assignments are confidential.
Seasonal context	The Q1 campaign represented regular plant

	operation. Incoming wood-chip moisture was typically about 25-35% before thermo-mechanical pulping. Ambient temperatures during the measurement windows were approximately -4.3 to 10.0 °C; relative humidity was approximately 36.5-93.9%.
Scrubber-water-temperature interpretation	A plant correlation check showed co-variation of scrubber-water temperature with throughput-related variables and dryer inlet temperature; therefore this variable is interpreted as an operational association rather than an isolated causal coefficient.
FTIR spectral-processing confidentiality	Original Q1 2022 spectra, proprietary spectral libraries, and reference-spectrum fitting outputs are not publicly disclosed. Later spectra were not used as direct evidence for the Q1 2022 dataset because they were not from the analysed campaign.
